# Metabolic reprogramming involving glycolysis in the hibernating brown bear skeletal muscle

**DOI:** 10.1186/s12983-019-0312-2

**Published:** 2019-05-06

**Authors:** Blandine Chazarin, Kenneth B. Storey, Anna Ziemianin, Stéphanie Chanon, Marine Plumel, Isabelle Chery, Christine Durand, Alina L. Evans, Jon M. Arnemo, Andreas Zedrosser, Jon E. Swenson, Guillemette Gauquelin-Koch, Chantal Simon, Stephane Blanc, Etienne Lefai, Fabrice Bertile

**Affiliations:** 1 Université de Strasbourg, CNRS, IPHC UMR 7178, F-67000 Strasbourg, France; 2 Department of Biology, Carleton University, Ottawa, ON K1S 5B6 Canada; 3 CarMen Laboratory, INSERM 1060, INRA 1397, University of Lyon, F-69600 Oullins, France; 4 Department of Forestry and Wildlife Management, Inland Norway University of Applied Sciences, Campus Evenstad, NO-2480 Koppang, Norway; 5 Department of Wildlife, Fish, and Environmental Studies, Swedish University of Agricultural Sciences, SE-901 83 Umeå, Sweden; 6 Department of Environmental and Health Studies, University College of Southeast Norway, N-3800 Bø, Telemark Norway; 7 Institute of Wildlife Biology and Game Management, University of Natural Resources and Life Sciences, A-1180 Vienna, Austria; 8 Faculty of Environmental Sciences and Natural Resource Management, Norwegian University of Life Sciences, NO-1432 Ås, Norway; 9 Norwegian Institute for Nature Research, NO-7485 Trondheim, Norway; 10 Centre National d’Etudes Spatiales, CNES, F-75001 Paris, France; 11 Université d’Auvergne, INRA, UNH UMR1019, F-63122 Saint-Genès Champanelle, France

**Keywords:** Hibernation, Brown bears, Skeletal muscle, Omics, Enzymology, Glycolysis, Lipid oxidation, Metabolism shift

## Abstract

**Background:**

In mammals, the hibernating state is characterized by biochemical adjustments, which include metabolic rate depression and a shift in the primary fuel oxidized from carbohydrates to lipids. A number of studies of hibernating species report an upregulation of the levels and/or activity of lipid oxidizing enzymes in muscles during torpor, with a concomitant downregulation for glycolytic enzymes. However, other studies provide contrasting data about the regulation of fuel utilization in skeletal muscles during hibernation. Bears hibernate with only moderate hypothermia but with a drop in metabolic rate down to ~ 25% of basal metabolism. To gain insights into how fuel metabolism is regulated in hibernating bear skeletal muscles, we examined the vastus lateralis proteome and other changes elicited in brown bears during hibernation.

**Results:**

We show that bear muscle metabolic reorganization is in line with a suppression of ATP turnover. Regulation of muscle enzyme expression and activity, as well as of circulating metabolite profiles, highlighted a preference for lipid substrates during hibernation, although the data suggested that muscular lipid oxidation levels decreased due to metabolic rate depression. Our data also supported maintenance of muscle glycolysis that could be fuelled from liver gluconeogenesis and mobilization of muscle glycogen stores. During hibernation, our data also suggest that carbohydrate metabolism in bear muscle, as well as protein sparing, could be controlled, in part, by actions of n-3 polyunsaturated fatty acids like docosahexaenoic acid.

**Conclusions:**

Our work shows that molecular mechanisms in hibernating bear skeletal muscle, which appear consistent with a hypometabolic state, likely contribute to energy and protein savings. Maintenance of glycolysis could help to sustain muscle functionality for situations such as an unexpected exit from hibernation that would require a rapid increase in ATP production for muscle contraction. The molecular data we report here for skeletal muscles of bears hibernating at near normal body temperature represent a signature of muscle preservation despite atrophying conditions.

**Electronic supplementary material:**

The online version of this article (10.1186/s12983-019-0312-2) contains supplementary material, which is available to authorized users.

## Background

Hibernation has evolved in a variety of mammalian species as an adaptive strategy to survive harsh winter environmental conditions, including seasonal cold ambient temperatures and food shortages [1, 2]. Hibernation/torpor expression implies trade-offs – on one hand physiological costs such as reduced memory retention, reduced immunocompetence and accumulation of sleep debt, and on the other hand, the major benefit of substantial fuel/energy conservation [3]. To save energy during the prolonged period of winter fasting, hibernators rely essentially on decreased metabolic rates over extended periods of deep torpor characterized by physical inactivity, reductions of heart and breathing rates and decreased body temperature [1]. Within this framework, the hibernation of bears represents an extreme phenotype that can last for up to 6-7 months [4] during which inactive animals do not eat, drink, urinate, defecate, or exhibit arousal episodes [5, 6]. Many aspects of bear physiology remain to be elucidated, including how their metabolism is regulated to enable them to endure such prolonged hibernation periods.

An average reduction in metabolic rate to 4.4% of normal basal rates and a decrease in body temperature down to about 6 °C have been calculated from the data available for 50-80 species of mammals that hibernate [2]. Relationships between reduced metabolic rate during hibernation and the body size and body temperature of hibernators have been nicely discussed elsewhere [7–9]. Metabolic rate and body temperature are clearly linked, but the dramatic reduction of metabolic rate has also been attributed to an active inhibition of many metabolic activities in a number of species [7–9]. However, the relative contributions of passive thermal effects (the so-called Arrhenius effect) versus active enzyme inhibition to metabolic suppression may differ among hibernators. Unlike the small mammal hibernators, such as rodents and bats of less than 10 kg [2], hibernating bears (family Ursidae; 80-100 kg [2]), maintain high body temperatures (32-35 °C), and the 75% decrease in bear metabolic rate that has been recorded is essentially achieved via active metabolic inhibition, independent of body temperature [5, 10, 11]. During hibernation, bears rely solely on body fuel reserves. Fat storage is increased prior to hibernation; e.g. Swedish brown bears (*Ursus arctos*) achieve this by notably overeating carbohydrate-rich berries [12]. Energy requirements are then met mainly through mobilization and oxidation of lipid fuels, bears losing 22-25% of body mass over the hibernating season [5, 13] with only a moderate loss of muscle protein [14, 15]. Accordingly, respiratory quotient (RQ) values as low as 0.62 to 0.73 have been recorded during denning [16]. However, the molecular mechanisms that are involved in organ/tissue metabolic adjustments have not yet been fully elucidated.

Skeletal muscle has a relatively low resting metabolic rate [17], but because it can account for as much as 40% of total body mass in humans [18], muscle is a major determinant of resting energy expenditure [19]. In addition, it has been reported that biochemical characteristics of skeletal muscle (i.e. in terms of enzymatic activities) are an important determinant of human whole-body metabolic rate and substrate oxidation [20]. Therefore, it could be proposed for hibernators that, as for the whole body, the metabolic fuel preference of skeletal muscle during hibernation is also lipids, whereas glucose oxidation is reduced. However, contrasting data exist about the regulation of fuel metabolism during hibernation, not only between small and large hibernators, but also between different studies of the same small mammal species. Whereas no coordinated transcriptional changes for genes involved in lipid catabolism has been recorded for the muscle of American black bears (*Ursus americanus*) [21], an increase in fatty acid oxidation during hibernation is supported by the upregulation of genes and proteins related to fatty acid catabolism in the muscle of hibernating thirteen-lined ground squirrels (*Ictidomys tridecemlineatus*) [22–25] and arctic ground squirrels (*Urocitellus parryii*) [26], as well as the muscle of Asiatic black bears (*Ursus thibetanus*) [27]. Concerning glucose metabolism, most of the available data support a decrease in muscle glycolysis during hibernation. Indeed, the recent application of transcriptomics technology has highlighted a coordinated under-expression of genes involved in glycolysis in skeletal muscles of both ground squirrels (*I. tridecemlineatus*, *U. parryii*) as well as *U. thibetanus* during hibernation [25–28]. In addition, it has been reported that protein and/or activity levels of hexokinase in muscle of Richardson’s ground squirrels (*Urocitellus richardsonii*) [29] and of glyceraldehyde-3-phosphate dehydrogenase in jerboa (*Jaculus orientalis*) [30] are reduced during hibernation. Consistently, proteomics analysis has shown diminished levels of glycolytic-related proteins in the muscle of winter thirteen-lined ground squirrels [22, 23]. Moreover, the kinetic characteristics of phosphofructokinase and fructose-1,6-bisphosphate aldolase at low temperatures could also be involved in suppressing glycolysis during hibernation, as suggested for the golden-mantled ground squirrel (*Callospermophilus lateralis*) [31, 32]. Finally, the link between muscle glycolysis and the tricarboxylic acid cycle appears to be disrupted during hibernation; for example, the mRNA and protein levels of PDK isozyme 4 (PDK4), which is known to inhibit pyruvate dehydrogenase (PDH) [33], are increased during hibernation in skeletal muscles of *I. tridecemlineatus* [34]. Conversely, other studies of this same species have reported results that may indicate maintenance of muscle glycolysis during hibernation. For example, carbohydrate-responsive element binding protein (ChREBP), a transcription factor activated by glucose metabolites, is more active in muscles of torpid squirrels [35]. Moreover, some kinetic features of muscle pyruvate kinase [36], the limited PDH regulation via phosphorylation [37], and the post-translational phosphorylation state of phosphoglucomutase (PGM1) [23] may also favour muscle glycolysis during hibernation.

The aim of the present study was to apply quantitative proteomics and biochemistry to brown bear skeletal muscle to test the hypothesis that changes in enzyme abundance and activity correspond to the expected decrease in metabolic rate and to determine if they could help to identify muscle fuel preferences (i.e. oxidation of lipid versus carbohydrate substrates). The results show that changes in enzyme activities do indeed appear to be relevant targets to gain insights into the biochemical mechanisms involved in metabolism suppression during hibernation. The metabolic reprogramming in muscles of hibernating brown bears does involve glycolysis although lipids remain the preferred fuels but with their rate of oxidation being reduced due to metabolic rate depression. Such regulations favour energy savings and the maintenance of muscle proteins in bears hibernating at a core body temperature that remains close to that of the summer-active period.

## Results

### Bear muscle proteome is dramatically changed during hibernation

**Fig. 1:**
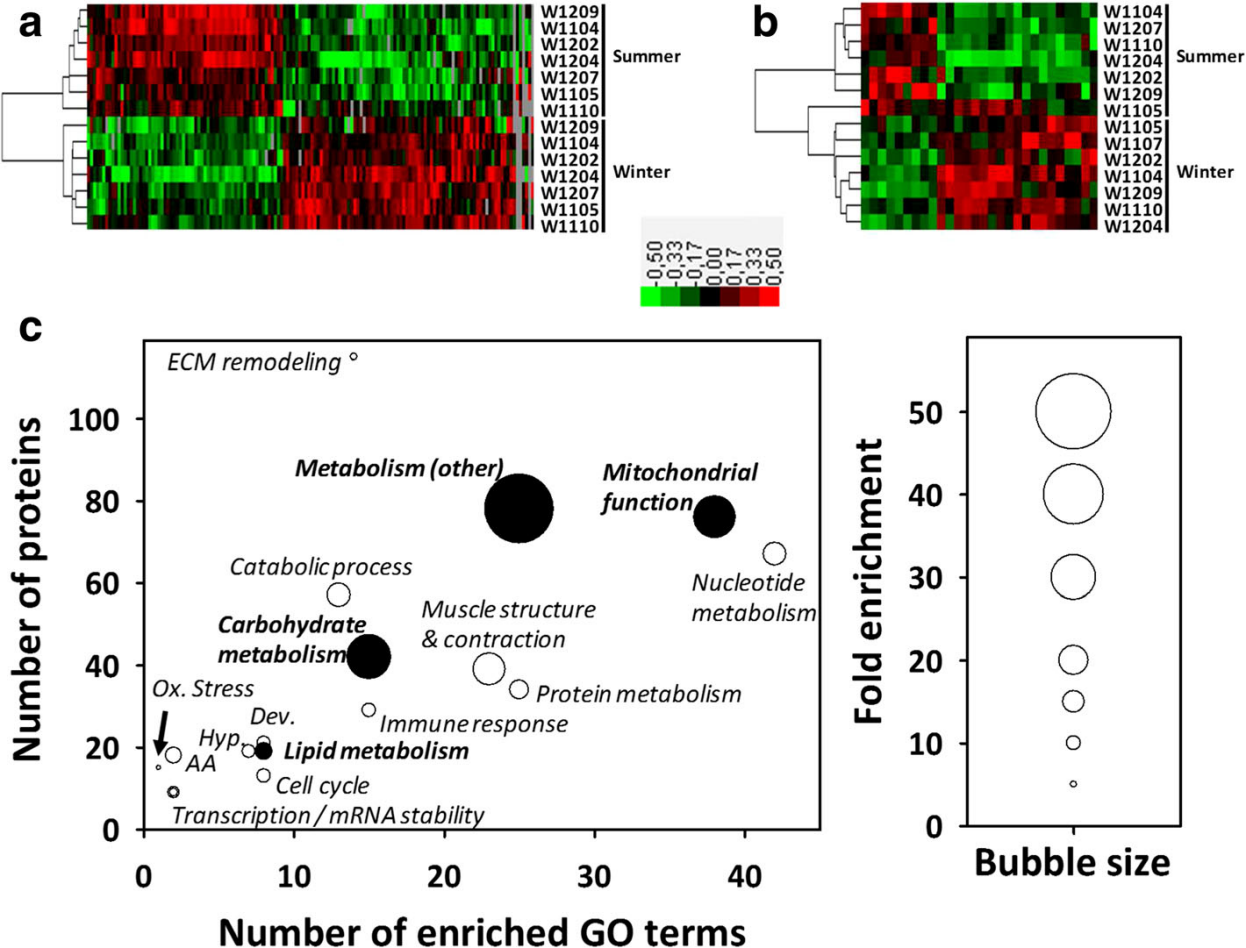
Overview of bear muscle proteomic response to hibernation. Changes in the proteome of brown bear vastus lateralis muscle between active (summer) and hibernating (winter) periods (*N* = 7 per season) are shown as heatmaps of differentially expressed proteins that were produced by hierarchical clustering from the MS1 quantitative-based (panel **a**) and 2D-DIGE-based (panel **b**) analyses. Signal values between animals from the two seasons were successfully discriminated (green, black and red boxes represent downregulated, intermediate and upregulated proteins, respectively). Functional annotation analysis from differential proteins revealed enriched Gene Ontology terms, which allowed determination of broad functions significantly affected by hibernation (panel **c**; filled circles represent the broad functions depicted by proteins that are discussed in this paper). Detailed protein abundances and fold changes are given in Additional file 1: Table S1 and Additional file 2: Table S2. AA: amino acid metabolism; Dev.: development & differentiation; ECM: extracellular matrix; Hyp.: response to hypoxia; Ox. Stress: response to oxidative stress

From label-free quantitative proteomics data (XIC), statistical analysis highlighted significant seasonal effects in the abundance of 146 muscle proteins, 67 of them being decreased and 79 increased in hibernating versus active bears (Fig. 1a, see also Additional file 1: Table S1).

Using two-dimensional difference in-gel electrophoresis (2D-DIGE), gel image analysis highlighted 28 protein spots (Additional file 3: Figure S1) exhibiting differential (*p* < 0.05) intensity in hibernating versus active bears (Additional file 2: Table S2). More precisely, the intensity of nine of these protein spots was lower in hibernating than active bears, whereas the reverse was observed for the remaining 19 spots (Fig. 1b). Mass spectrometry allowed 62 different proteins to be identified in these 28 protein spots, including 47 proteins that were considered as ‘major proteins’ (i.e. more abundant in given spots; see Methods section) and thus purportedly responsible for the changes in protein spot intensities (Additional file 2: Table S2).

Importantly, the overlap of quantitative data between the two proteomics approaches was highly consistent, as all of the eight commonly identified proteins exhibited very similar seasonal effects (Additional file 2: Table S2). From a merged list of differentially-expressed proteins coming from the two proteomics approaches, functional annotation analysis revealed that differences between hibernating and active bears involved mostly proteins known to play roles in muscle metabolism in a broad sense and in structural remodelling (Fig. 1c). Based on these data, we decided to focus our attention and extend the analysis for a more in-depth examination of muscle fuel metabolism.

### Bear muscle proteomics highlights the regulation of fuel metabolism during hibernation

**Fig. 2:**
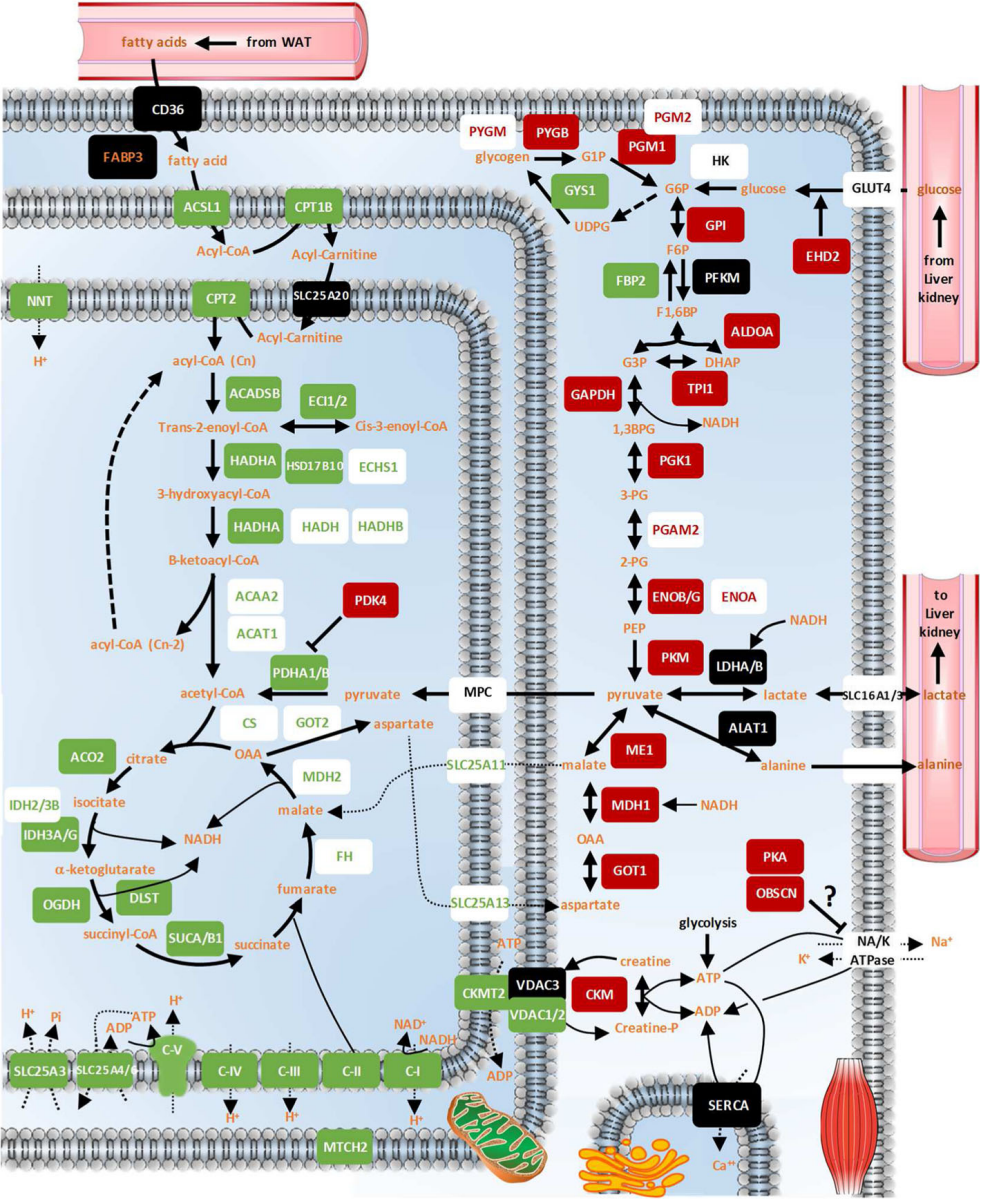
Regulation of metabolism-related factors in hibernating bear muscles. The relative abundance of muscle (vastus lateralis) proteins in winter (hibernating) versus summer (active) brown bears (N = 7 per season) is shown using the following colour code: significantly (Welch student and paired Student tests; *P* < 0.05) up- and down-regulated proteins are shown in red and green boxes, respectively; black boxes show proteins that did not change, and white boxes show proteins that were not detected (in black letters) or those for which slightly less reproducible up- (in red) or down- (in green) regulation was recorded. FABP3 (in orange) was identified in two distinct protein spots (2D-DIGE strategy) that exhibited opposite responses, suggesting the possible occurrence of post-translational modifications. Detailed protein abundances and fold changes are given in Additional file 1: Table S1 and Additional file 2: Table S2. WAT: white adipose tissue

We achieved a nearly comprehensive detection of the muscle proteins involved in lipid oxidation, glycolysis, and major ATP production (mitochondrial respiratory chain) and consumption (e.g. ion pumping Na^+^/K^+^ and Ca^2+^ ATPases) processes. The data supporting the changes described below are presented in Fig. 2 and Additional file 1: Table S1 and Additional file 2: Table S2.

Regarding muscle lipid metabolism, the expression levels of only two proteins remained essentially unchanged in winter versus summer bears, namely fatty acid translocase (CD36) and mitochondrial carnitine/acylcarnitine carrier protein (SLC25A20). Fatty acid binding protein 3 (FABP3) was detected in two different protein spots on 2D-DIGE gels, one exhibiting significantly lower (1.3 times) and the other higher (1.2 times) intensity in hibernating versus active bears. All other proteins involved in fatty acid beta-oxidation were found at significantly lower levels during hibernation, including long chain fatty acid-CoA ligase 1 (ACSL1; 2.2 times), carnitine O-palmitoyltransferases 1 (CPT1B; 3.4 times) and 2 (CPT2; 1.2 times), short/branched chain specific acyl-CoA dehydrogenase (ACADSB; 1.5 times), enoyl-CoA delta isomerases 1 (ECI1; 1.5 times) and 2 (ECI2; not detectable in winter), trifunctional enzyme subunit alpha (HADHA; 1.3 times) and beta (HADHB; 1.4 times), 3-hydroxyacyl-CoA dehydrogenase type-2 (HSD17B10; 1.6 times), enoyl-CoA hydratase (ECHS1; 1.5 times), hydroxyacyl-CoA dehydrogenase (HADH; 1.4 times), 3-ketoacyl-CoA thiolase (ACAA2; 1.5 times), and acetyl-CoA acetyltransferase (ACAT1; 1.6 times).

Similarly, all the proteins of the tricarboxylic acid cycle were downregulated in hibernating bear muscle, including citrate synthase (CS; 1.5 times), aconitate hydratase (ACO2; 1.5 times), isocitrate dehydrogenase (IDH2; 1.4 times) and isocitrate dehydrogenase subunits alpha, (IDH3A; 1.8 times), beta (IDH3B; 1.7 times) and gamma (IDHG; 2 times), 2-oxoglutarate dehydrogenase (OGDH; 1.4 times), succinyl-CoA ligase subunits alpha and beta (SUCA and SUCB1; 1.5 times), fumarate hydratase (FH; 1.5 times), and malate dehydrogenase (MDH2; 1.4 times).

Mitochondrial membrane respiratory chain protein complexes I to V are each composed of multiple different subunits that were similarly downregulated in hibernating bear muscles, including several subunits of NADH dehydrogenase (NDUFA9, NDUFA10, NDUFB9, NDUFB10, NDUFS1, NDUFS2, NDUFS3, NDUFS8, NDUFV1, NDUFV2; 1.4-2.1 times; as well as MT-ND5 not detectable in winter), succinate dehydrogenase (SDHA and SDHB; 1.3-1.4 times), cytochrome c reductase (UQCRC2, CYC1 and UQCRFS1; 1.3-1.5 times), cytochrome c oxidase (COX2 and COX5A; 1.4-2.7 times), and ATP synthase (ATP5A1, ATP5B, ATP5C1, ATP5F1, ATP5H and ATP5O; 1.2-2.7 times). Six other NADH dehydrogenase subunits, one other subunit of cytochrome c reductase, and two other ATP synthase subunits were detected and also showed a reduced trend in muscle of hibernating bears, although differences were not statistically significant. Levels of other proteins closely related to the mitochondrial respiratory chain and/or membrane potential were also reduced in winter bears, including phosphate carrier protein (SLC25A3; 1.9 times), ADP/ATP translocases 1 and 3 (SLC25A4 or ANT1 and SLC25A6 or ANT3; 1.4-1.7 times), NAD(P) transhydrogenase (NNT; 2 times), and mitochondrial carrier homolog 2 (MTCH2; 1.6 times). Finally, the abundance of mitochondrial creatine kinase was decreased during hibernation (CKMT2; 1.5 times), as well as that of voltage-dependent anion-selective channel proteins 1 and 2 (VDAC1 and VDAC2; 1.5 times), whereas voltage-dependent anion-selective channel protein 3 (VDAC3) remained unchanged.

Glycogen metabolism proteins were regulated during hibernation, with decreased levels of glycogen synthase (GYS1; 1.3 times) but increased levels of glycogen phosphorylase (PYGM and PYGB; 1.2-1.8 times) and phosphoglucomutase-1 and -2 (PGM1 and PGM2; 1.3-1.4 times). Concerning glycolysis, only levels of ATP-dependent 6-phosphofructokinase (PFKM) remained stable between winter and summer bears. The levels of all other proteins were found to increase during hibernation, including those of EH domain-containing protein 2 (EHD2; 1.6 times), glucose-6-phosphate isomerase (GPI; 1.4 times), fructose-bisphosphate aldolase A (ALDOA; 1.3 times), triosephosphate isomerase (TPI1; 1.2 times), glyceraldehyde-3-phosphate dehydrogenase (GAPDH; 1.2 times; *P* = 0.052), phosphoglycerate kinase 1 (PGK1; 1.2 times), phosphoglycerate kinase 2 (PGAM2; 1.4 times), alpha-, beta- and gamma-enolase (ENOA, ENOB and ENOG; 1.3-1.4 times), and pyruvate kinase (PKM; 1.3 times). One protein of gluconeogenesis was detected, namely fructose-1,6-bisphosphatase isozyme 2 (FBP2), with decreased levels (1.2 times) in skeletal muscle of hibernating animals.

Examining the different pathways of pyruvate metabolism, we observed decreased levels of pyruvate dehydrogenase E1 component subunits alpha and beta (PDHA1 and PDHB; 1.4 times) in winter versus summer bears, whereas increased levels were recorded for pyruvate dehydrogenase kinase isozyme 4 (PDK4; not detectable during summer) and malic enzyme (ME1; 2 times). The levels of lactate dehydrogenase A and B chains (LDHA and LDHB) and of alanine aminotransferase 1 (ALAT1) were not significantly different between seasons. In addition, we observed upregulation of cytosolic malate dehydrogenase (MDH1; 1.3 times) and aspartate aminotransferase (GOT1; 1.3 times) and downregulation of mitochondrial 2-oxoglutarate/malate carrier protein (SLC25A11; 1.6 times), calcium-binding mitochondrial carrier protein Aralar2 (SLC25A13; 1.4 times), and mitochondrial aspartate aminotransferase (GOT1; 1.3 times) in hibernating bears.

The levels of cytosolic creatine kinase were increased during winter (CKM; 1.2 times). Of the main ion pumps of muscle cells, Na^+^/K^+^ ATPase was not detected, but the levels of obscurin were increased during hibernation (OBSCN; 1.7 times), as were those of cAMP-dependent protein kinase A (PKA) subunits (PRKAR1A and PRKAR2A; 2.1-2.6 times, and PRKACA; 1.3 times with *P* = 0.06). The levels of three isoforms of sarcoplasmic/endoplasmic reticulum Ca^2+^ ATPase (SERCA1-3) remained stable between winter and summer bears.

### Bear muscle proteome changes were validated by quantitative RT-qPCR, western-blot and enzyme activity assays

**Fig. 3:**
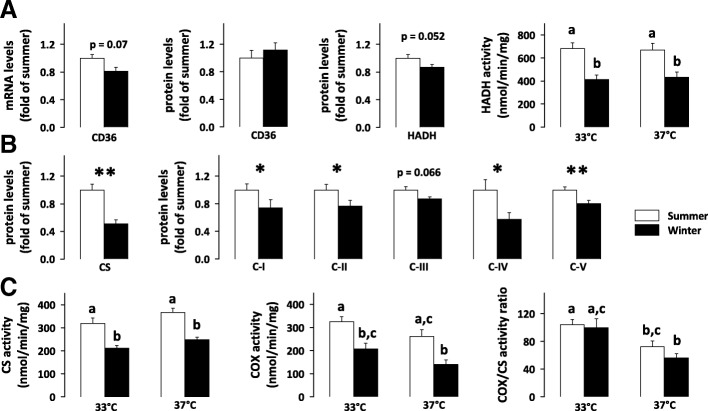
Repression of lipid metabolism and mitochondrial oxidative phosphorylation in winter bear muscles. Protein or mRNA expression levels of fatty acid translocase (CD36), mitochondrial hydroxyacyl-coenzyme A dehydrogenase (HADH), citrate synthase (CS) and subunits of the five oxidative phosphorylation (OXPHOS) complexes (C-I to C-V) were measured using RT-qPCR and/or Western-blot analysis in vastus lateralis muscle samples from brown bears in summer (white bars) and winter (black bars) (panels **A** and **B**; *N* = 8-12 per season). Corresponding blots are shown in Additional file 4: Figure S2. Maximum enzymatic activities (panels **A** and **C**) for HADH, CS and cytochrome c oxidase (COX) were measured at 33 °C and 37 °C (N = 7 per season). Data are expressed as means ± sem. Statistical significance is shown for paired student t-tests (* *P* < 0.05; ** *P* < 0.01) or post-hoc Tukey tests that followed type III ANOVA (values that do not share the same superscript letter are significantly different; *P* < 0.05)

Regarding lipid metabolism, the levels of fatty acid translocase (CD36) remained unchanged between winter and summer bears, both at the mRNA and protein levels (Fig. 3A and Additional file 4: Figure S2). The decrease in hydroxyacyl-CoA dehydrogenase (HADH) protein levels during winter was not statistically significant. However, HADH enzymatic activity was significantly reduced in hibernating bears, with a similar 1.5-1.7-fold magnitude, regardless of whether the temperature at which reaction was carried out was 33 °C or 37 °C, to mimic the mean body temperature of bears during winter and summer, respectively.

Concerning the tricarboxylic acid cycle and mitochondrial respiratory chain, significantly decreased protein levels of citrate synthase (CS; 2 times) and subunits of mitochondrial complexes I, II, IV and V (1.3-1.7 times) were observed in the skeletal muscle of hibernating bears (Fig. 3B and Additional file 4: Figure S2). Consistently, enzymatic activity levels of CS and cytochrome c oxidase (COX, complex IV) were also reduced significantly by 1.5-1.9 times during winter, when performing the reaction at either 33 °C and 37 °C (Fig. 3C). As a consequence, the COX/CS activity ratio was not different between winter and summer. When activity assays were performed at 37 °C, the COX/CS activity ratio was nevertheless significantly lower than when activity assays were performed at 33 °C (1.4 times for summer samples and 1.8 times for winter samples).

**Fig. 4:**
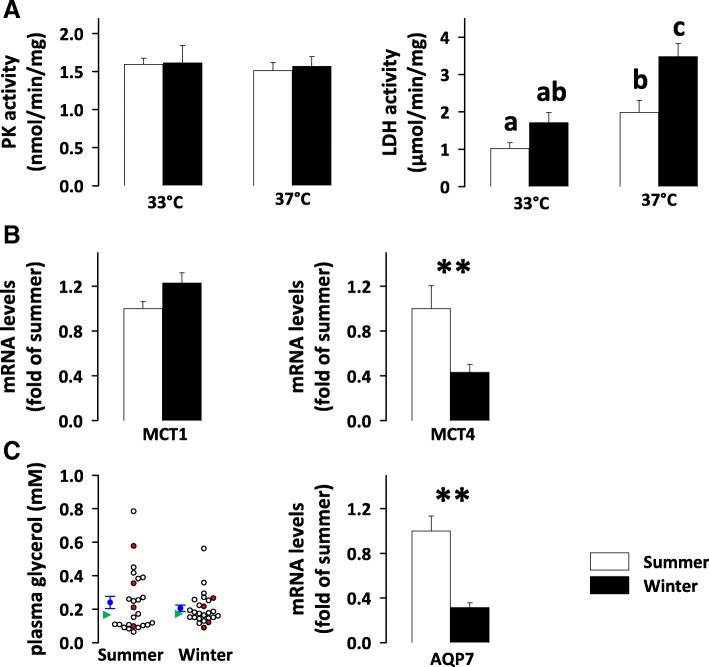
Changes related to carbohydrate metabolism in winter bears. Maximum enzymatic activities of pyruvate kinase (PK) and lactate dehydrogenase (LDH) were measured in vastus lateralis muscle samples from brown bears in summer (white bars) and winter (black bars) at 33 °C and 37 °C (panel **A**; N = 7 per season). Gene expression levels of monocarboxylate transporter 1 (MCT1 or SLC16A1), monocarboxylate transporter 4 (MCT4 or SLC16A3), and aquaporin 7 (AQP7) were measured using RT-qPCR in bear vastus lateralis muscles (panel **B** and C; N = 8 per season). Data are expressed as means ± sem. Statistical significance is shown for paired student t-tests (** *P* < 0.01) and post-hoc Tukey tests that followed type III ANOVA (values that do not share the same superscript letter are significantly different; *P* < 0.05). Circulating levels of glycerol were assessed enzymatically in bear plasma using a commercial kit (panel **C**; *N* = 25 per season) and presented as individual values (circles, those in red correspond to plasma samples where glycerol was also measured using NMR-based analysis with values being shown in Table 1) along with the mean ± sem (in blue) and median (in green) values

Concerning glycolysis, pyruvate kinase activity in bear muscle remained stable across seasons, when measured at either 33 °C or 37 °C (Fig. 4A). Enzymatic activity levels of lactate dehydrogenase (LDH), which catalyses pyruvate conversion to lactate, were found to be increased in hibernating bears, not significantly when the reaction was performed at 33 °C but with a significant 1.8-fold change at 37 °C (Fig. 4A). Gene expression levels of membrane lactate transporters (Fig. 4B) were either unchanged between seasons (monocarboxylate transporter 1, MCT1 or SLC16A1) or significantly decreased during winter (monocarboxylate transporter 4, MCT4 or SLC16A3; 2.3 times). Finally, we also oberved a decreased gene expression of aquaporin 7 (AQP7; 3.2 times) in hibernating bear muscle, although in the aquaporin family, this isoform actually has a major role as a glycerol transporter (Fig. 4C).

### Bear plasma metabolite profiles are changed during hibernation

Plasma metabolite measurements assayed via enzymatic kits and those derived from nuclear magnetic resonance (NMR) analysis were in good agreement for glucose and lactate levels (Table 1). Thus, glycaemia tended to be higher in winter compared to summer, but the difference did not reach significance. Oppositely, lactate levels were significantly reduced in hibernating bear plasma (1.4-2 times). Free fatty acid levels were increased during hibernation (2.7 times) (Table 1). Concerning glycerol levels, the significant decrease (1.3 times) in winter, measured using NMR-based analysis, was also found using the enzymatic kit when considering exactly the same samples (see Table 1 and the red dots in Fig. 4C). Because the values were markedly variable among individuals, especially in summer, we extended the measurement of glycerol levels to 25 bears and still observed no significant seasonal effect (Table 1 and Fig. 4C). We also observed significantly higher circulating levels of triacylglycerols (1.3 times), 3-hydroxybutyrate (1.7 times), betaine (1.4 times), and creatinine (1.5 times) in hibernating bears. No significant change was recorded for plasma levels of pyruvate, glycine/sarcosine, and myo-inositol.

**Table 1:** Brown bear plasma metabolite profiles

	Summer	Winter	p-value	N/season	
Plasma metabolite assays using enzymatic methods (kits)					
Glucose (mM)	4.10 ± 0.83	5.90 ± 0.41	0.0913	6	–
Free fatty acids (mM)	0.14 ± 0.02	0.38 ± 0.03	**0.0003**	7	–
Lactate (mM)	8.59 ± 1.31	4.28 ± 0.95	**0.0125**	7	
Glycerol (mM)	0.24 ± 0.04	0.20 ± 0.02	0.3944	25	–
Triacylglycerols (mM)	2.85 ± 0.20	3.60 ± 0.31	**0.0124**	6	–
Plasma NMR-based analysis					mean VIP
Glucose (au)	1.00 ± 0.07	1.21 ± 0.05	0.0598	7	1.73
Unidentified fatty acid (au)	1.00 ± 0.15	1.25 ± 0.15	0.3659	7	2.19
Lactate (au)	1.00 ± 0.09	0.72 ± 0.08	0.0672	7	1.70
Glycerol (au)	1.00 ± 0.04	0.78 ± 0.03	**0.0059**	7	1.05
3-hydroxybutyrate (au)	1.00 ± 0.06	1.65 ± 0.10	**0.0007**	7	1.86
Pyruvate (au)	1.00 ± 0.10	1.22 ± 0.10	0.2561	7	1.21
Glycine/sarcosine (au)	1.00 ± 0.06	0.82 ± 0.04	0.0606	7	1.02
Glutamine (au)	1.00 ± 0.06	1.19 ± 0.09	0.1768	7	1.02
Myo-inositol (au)	1.00 ± 0.20	1.40 ± 0.24	0.2155	7	2.47
Betaine (au)	1.00 ± 0.08	1.37 ± 0.13	**0.0244**	7	1.90
Creatinine (au)	1.00 ± 0.06	1.51 ± 0.15	**0.0126**	7	1.38
Plasma liquid chromatography-based analysis of amino acids					
Alanine (**μ**M)	597.8 ± 55.1	441.1 ± 31.7	**0.0234**	8	–
Glutamine (**μ**M)	479.4 ± 54.0	631.1 ± 31.3	**0.0356**	8	–

Data are the means ± SEM of indicated individual determinations in hibernating (winter; February) versus active (summer; June) brown bears (*Ursus arctos*). Significant differences between summer and winter (*p*-values < 0.05), are highlighted in bold

Concerning plasma amino acids (Table 1), although the 1.2-fold increase in glutamine levels measured using NMR-based analysis was not significant, specific measurements using liquid chromatography-based assay indicated a significant 1.3-fold increase in glutamine levels during the hibernation period. On the contrary, the levels of plasma alanine were significantly decreased (1.4 times) in hibernating bears.

### Bear muscle glycogen content and serum fatty acid concentrations are changed during hibernation

**Fig. 5:**
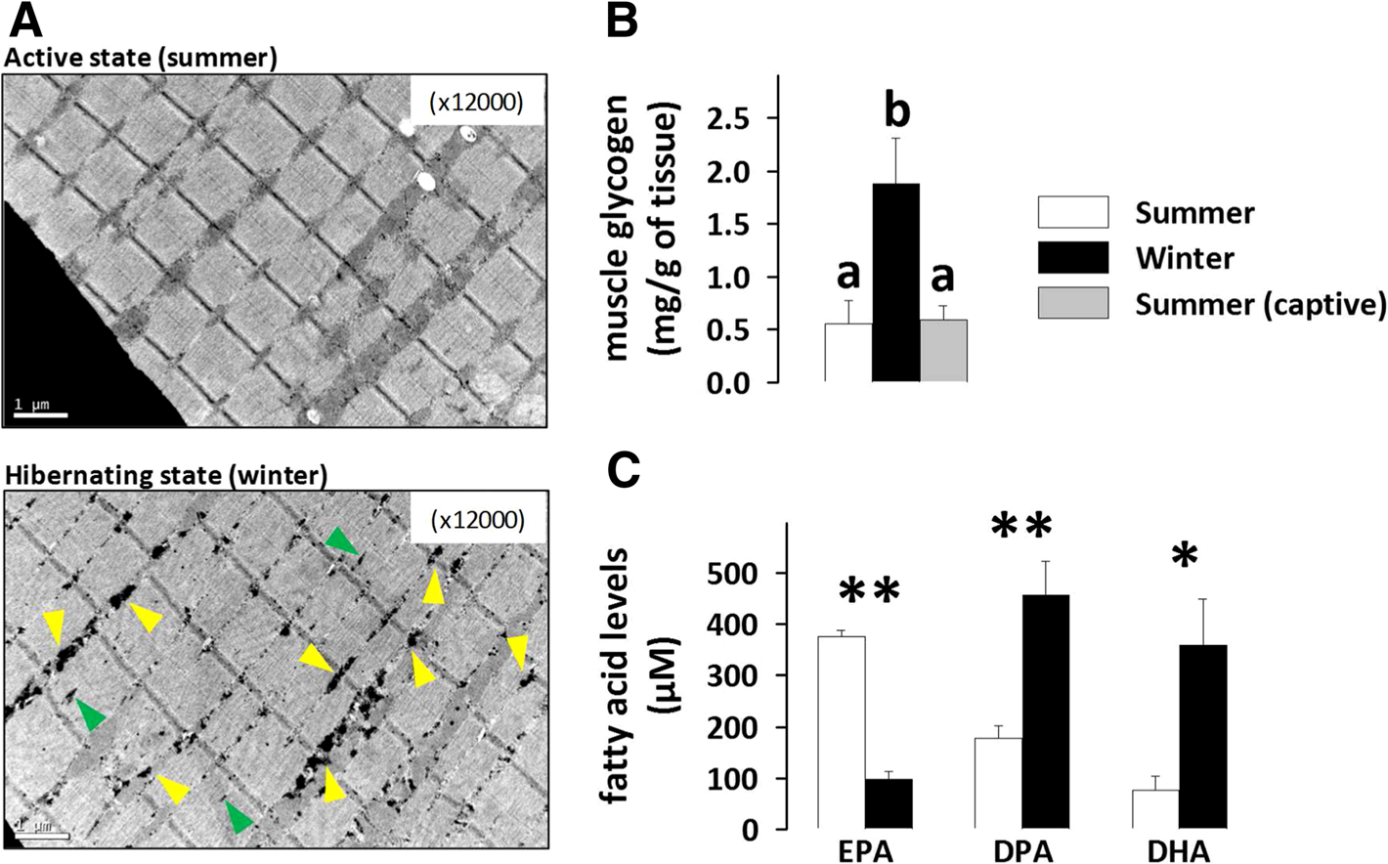
Muscle glycogen content is increased and serum fatty acid profiles are changed in winter bear muscles. As illustrated on representative electron micrographs of brown bear vastus lateralis muscle (panel **A**), intramyofibrillar (green) and especially intermyofibrillar (yellow) glycogen granules accumulate in skeletal muscle of hibernating bears (lower panel), whereas their presence was largely undetectable in active summer bears (upper panel) at this magnification level. Glycogen content was measured in bear muscles (panel **B**; N = 7 per group). Circulating levels of eicosapentaenoic acid (EPA), docosapentaenoic acid (DPA) and docosahexaenoic acid (DHA) were assessed in bear serum (panel **C**; *N* = 4 serum mixes per season). Data are expressed as means ± sem. Statistical significance is shown for paired student t-tests (* P < 0.05; ** *P* < 0.02) and post-hoc Tukey tests that followed one-way ANOVA (values that do not share the same superscript letter are significantly different; P < 0.05)

Strikingly, the skeletal muscle glycogen content observed on electron micrographs was higher in hibernating than active bears (Fig. 5A). Assaying muscle glycogen content confirmed this observation, with a significant ~ 3 times higher level of glycogen in muscle of hibernating bears compared to summer active animals (Fig. 5B). Because muscle glycogen rapidly becomes the most important substrate source to sustain rapid muscle movement during physical exercise [38] and summer bears ran for several minutes during helicopter darting, we hypothesized that this could have caused a lower level of glycogen in their muscle. However, the similar values we measured in summer captive animals, which did not run prior to sampling, invalidated this hypothesis and indicated that the short run of wild summer bears was not sufficient to provoke significant muscle glycogen depletion.

Lipidomics analysis of polyunsaturated fatty acids (PUFA) highlighted significantly reduced levels of eicosapentaenoic acid (20:5n-3; EPA; 3.8 times) and increased levels of docosapentaenoic acid (22:5 n-3; DPA; 2.6 times) and docosahexaenoic acid (22:6 n-3; DHA; 4.7 times) in the serum of hibernating versus active bears (Fig. 5C).

## Discussion

Hibernation is a physiological fasting state where physical inactivity, metabolic rate depression, and a decrease in core body temperature allow for effective fuel and energy savings sufficient to sustain survival over the winter months. In such a metabolic context, energy supply appears to come primarily from lipid substrate oxidation, whereas glucose oxidation is reduced. However, contrasting data exist about the utilization of lipid versus carbohydrate fuels in the muscle of hibernators (see above in the Introduction section). Our data show that expression and activity levels of lipid oxidizing enzymes were lower in hibernating brown bears, whereas the expression levels of muscle glycolysis enzymes were higher during hibernation (see Fig. 2 and Additional file 1: Table S1 and Additional file 2: Table S2).

### ATP production and consumption are tuned down in hibernating bear skeletal muscle

As an energy-saving adaptive strategy, hibernation is characterized by low metabolic rates [2]. As a consequence, metabolic fuel consumption and ATP turnover should be decreased throughout the body of hibernators, and in their muscles in particular.

The general decrease in protein abundance of subunits of the five complexes of the mitochondrial respiratory chain, measured here in muscles of hibernating brown bears (Figs. 2 and 3), extends transcriptomics data previously reported in hibernating American black bears (*Ursus americanus*) [28]. These regulations could lead to a reduced capacity for ATP production in skeletal muscles of hibernating bears, which could be linked to the reduced energy demand during the hibernation period.

Since the functional coupling between oxidative phosphorylation and the mitochondrial creatine kinase system has been previously reported [39], it was not surprising to see the generally reduced expression levels of CKMT2, as well as of associated proteins like the porins VDAC1 and 2 and ADP/ATP translocases (SLC25A4 and 6). Interestingly, mitochondrial cytochrome c oxidase (COX, complex IV) activity was lower in muscles of hibernating versus active bears, independent of assay temperature (Fig. 3). This decreased activity of COX could be related to the decreased amount of muscle COX protein during hibernation. These results further support an overall reduction in muscle ATP synthesis capacity in hibernating bears.

A decrease in abundance of all proteins of the tricarboxylic acid cycle along with a reduction in citrate synthase (CS) activity levels was also seen in bear skeletal muscle in the winter, likely reflecting a lower mitochondrial content. From samples collected in a given period (i.e. summer or winter), the assay temperature did not affect CS nor COX activity levels, which were quantified as maximal velocity (Vmax). These results are in line with the fact that the COX to CS activity ratio was not different between seasons, independent of assay temperature, and suggest that the intrinsic maximal oxidative capacity of winter mitochondria does not differ from that of summer mitochondria. This is consistent with previous results showing either a modest reduction in the respiration rates of mitochondria isolated from skeletal muscle of thirteen-lined ground squirrels during torpor compared with interbout euthermia [40] or no change in mitochondria from muscles of hibernating versus summer-active arctic ground squirrels [41]. We observed higher COX/CS values in bears when assays were performed at 33 °C than 37 °C. At winter body temperature, this could enable skeletal muscles to rapidly increase their ATP output whenever muscle work is required, e.g. if the integrity of the hibernaculum is no longer maintained in the middle of winter and the bear must find a more safe and suitable den. Altogether, these results indicate that suppression of bear muscle mitochondrial respiration during hibernation likely results in a decreased mitochondrial content and suppressed electron supply to the respiratory chain.

Ion pumping through the Na^+^/K^+^ and Ca^2+^ ATPases is a major ATP-consuming process in mammal cells [42]. In muscles of golden-mantled ground squirrels, reduced levels of Na^+^/K^+^ ATPase activity during hibernation were reported to possibly be due to phosphorylation by cAMP-dependent protein kinase A (PKA) [43]. We did not detect subunits of the Na^+^/K^+^ ATPase in either summer-active or in hibernating bear muscles. However, we observed increased levels of PKA during the winter that could potentially be involved in repressing activity of bear muscle Na^+^/K^+^ ATPase, and other enzymes that are sensitive to PKA regulation, during hibernation (Fig. 2). In addition, obscurin (OBSCN) has been shown to interact with Na^+^/K^+^ ATPase [44], and, although its ability to phosphorylate Na^+^/K^+^ ATPase has not be demonstrated in vitro, its increased levels in muscle of hibernating bears make it a possible candidate for modulating ion pump activity. Sarcoplasmic/endoplasmic reticulum Ca^2+^ ATPase (SERCA) has also been shown to be repressed, both in activity and protein levels in skeletal muscle of the long-tailed ground squirrel (*Urocitellus undulatus)* during winter [45]. We found that the abundance of the three SERCA isoforms did not change in the muscle of hibernating bears, but we cannot exclude the possibility that SERCA activity could be modulated during hibernation such as by endogenous protein effectors like sarcolipin, by post-translational modifications, or in relation with the lipid composition of the sarcoplasmic reticulum membrane [46, 47].

Reactions of membrane ion pumping systems that imply ATP consumption are generally connected to those catalysed by enzymes producing ATP, e.g. creatine kinase [39]. We observed that cytosolic creatine kinase (CKM) was only slightly more abundant in skeletal muscle of hibernating bears and that mitochondrial CKMT2 levels were reduced. Whether this could modulate creatine/phosphocreatine metabolism remains to be determined. Connected to the metabolism of creatine is that of creatinine [48], and increased plasma creatinine reflects its diminished renal clearance in hibernating brown bears.

Protein synthesis is also an important process that consumes ATP [42]. We know that protein synthesis rates are dramatically decreased in muscles of hibernating bears [14], thus favouring energy sparing. Because betaine has positive effects on lean muscle mass [49], its increased levels in the plasma of hibernating bears may possibly contribute to reducing muscle protein loss (i.e. minimizing atrophy).

Altogether, our data suggest that ATP production and consumption are turned down in skeletal muscle of hibernating bears, which would help to reduce metabolic rate, while maintaining metabolic balance. To go further, future studies should assess and compare skeletal muscle mitochondrial respiration in hibernating versus summer active bears and examine muscle fiber and mitochondrial responses to various energy substrates in more detail.

### Lipid oxidation is reduced, but preferred, in hibernating bear skeletal muscle

It is commonly recognized that lipids are the main energy fuel during hibernation. In this situation, triacylglycerols from fat stores are hydrolysed and fatty acids are released into the blood flow. Accordingly, we found that the amounts of total free fatty acids and those of 3-hydroxybutyrate were both elevated in the plasma of hibernating bears, a result consistent with previous studies [50]. This increased availability of circulating lipid fuels offers fat-burning tissues, like skeletal muscle, the possibility to enhance lipid uptake and oxidation, thereby sparing the much more limited reserves of glycogen and glucose.

The transport of circulating fatty acids into muscle cells is facilitated by fatty acid translocase (CD36). Our findings, that the levels of muscle CD36 mRNA and protein remained stable in the bears between summer and winter (Figs. 2 and 3), would suggest that fatty acid entry into muscle cells is not affected by hibernation, although we cannot be conclusive on that point. Once in the cells, fatty acids traffic towards the mitochondria via transport by fatty acid binding proteins (FABP), especially FABP3 in muscle. In the muscle of ground squirrels, upregulation of FABP3 has been reported at both transcript [24, 25] and protein [23] levels during hibernation. Here we identified bear FABP3 in two different protein spots on 2D-DIGE gels, which exhibited a different regulation. This suggests a post-translational regulation of bear FABP3 during hibernation; however we were not able to determine the nature of the post-translational modification at play. To our knowledge, it is not known whether post-translational modifications of FABP3 alter its functions. In particular, we do not know whether such regulation could be linked to its role in fatty acid trafficking and/or possible role in stimulating glucose uptake [51] (see below).

Increased expression of several beta-oxidation enzymes has been reported in the skeletal muscle tissue of hibernating ground squirrels at both the mRNA [25] and protein [22, 23] levels. In black bears, muscle data were available only for the mRNA level and showed either no change [21] or an increase [27] in expression of fatty acid catabolism-related genes during hibernation. Opposite to these results, we found that abundance of all proteins of the beta-oxidation pathway was  decreased in the muscle of brown bears during the hibernation period, except SLC25A20, which remained unchanged (Figs. 2 and 3). The decreased activity of hydroxyacyl-CoA dehydrogenase (HADH) that we recorded in muscles of hibernating bears further supports a slowing of the beta-oxidation pathway. Such a globally coordinated downregulation of fatty acid catabolizing capacity accords well with lowered TCA and respiratory chain rates proposed above for hibernating bear muscle. The discrepancy with changes in mRNA levels in black bear muscles may reflect a drop in translational regulation, rather than an increase in transcription rates, in line with globally lower protein synthesis rates in hibernating bear muscles [14]. By contrast, the upregulated expression of beta-oxidation factors in the muscle of hibernating ground squirrels [22, 23] could be a mechanism that helps to sustain a certain level of activity despite very low body temperatures (often near 0 °C) while in hibernation.

Although we argue above for a diminished capacity for oxidation of fatty acids, we do not question the bear’s reliance on lipids as the main fuel source for muscle ATP generation during hibernation. Indeed, as is the case for hibernating thirteen-lined ground squirrels [34], a disruption of carbohydrate use for fuelling the TCA cycle also appears to occur in hibernating bears, as corroborated by the upregulation of pyruvate dehydrogenase kinase isoenzyme 4 (PDK4) and concomitant downregulation of pyruvate dehydrogenase (PDHA1 and B).

### Glycolysis appears to be maintained in hibernating bear skeletal muscle

During hibernation in animals such as ground squirrels, contrasting data have been reported concerning muscle oxidation of glycolytic intermediates. A number of studies have reported data supporting a decrease in glycolysis rate with downregulation of several glycolytic enzymes, at the mRNA [25, 26, 28], protein [22, 23] or activity [29] levels. Others studies have reported, on the contrary, that the post-translational regulation of PDH and PGM1 would favour glycolysis maintenance during hibernation in thirteen-lined ground squirrels [23, 37]. In previous studies of hibernating bears, only results at the mRNA level were available, which showed reduced expression of several glycolytic genes [27, 28]. Our results (Figs. 2 and 4) show a global increase in the abundance of all glycolytic enzymes in the skeletal muscle of hibernating brown bears, except for GLUT4 and hexokinase (that were not detected) and ATP-dependent 6-phosphofructokinase (PFKM; unchanged). Such adjustments would be in line with a possible increase in the capacity of hibernating bear muscle for glycolysis, or at least its maintenance, if we also consider the unmodified activity of pyruvate kinase (PKM) that we observed between seasons. Taking into account the above-mentioned block in the mitochondrial conversion of pyruvate to acetyl-CoA, it could be hypothesized that glycolysis is oriented more toward lactate production in hibernating brown bear skeletal muscles. Interestingly, previous data have shown that anaerobic glycolysis is increased in cardiac muscle of ground squirrels during hibernation [52]. Absolute values for LDH activity are lower when the assay is performed at 33 °C versus 37 °C. However, the activity of LDH measured at 37 °C in summer muscles was not significantly different from that of winter muscles measured at 33 °C. This may reflect a certain maintenance of LDH activity, which could help maintain the capacity for anaerobic glycolysis during hibernation. In skeletal muscles, lactate transport across the plasma membrane is essentially done via monocarboxylate transporters 1 and 4 (SLC16A1 and SLC16A3). Decreased gene expression of SLC16A3 may be another clue that anaerobic glycolysis is regulated in hibernating bear muscles. Comparing lactate levels in skeletal muscles of winter and summer bears in future studies will help to better understand the glycolytic flux during hibernation.

Hence, our results suggest that glycolytic capacity is retained in muscle, which could allow a rapid muscle response if the bear is disturbed and arouses.

### What are the possible sources of glucose during hibernation in bears?

If glycolytic capacity is retained in skeletal muscle of hibernating bears, an adequate availability of glycolytic substrates should be ensured during hibernation. Accordingly, plasma glucose levels tended to be increased during hibernation in our study and statistical significance has even been reached in previous studies [50]. During hibernation, glucose can be derived from mobilization of glycogen stores and from gluconeogenesis that occurs mainly in the liver. The main substrates for liver gluconeogenesis are glycerol, lactate, and alanine. Although glycerol is released by white adipose cells due to lipolysis and although muscle lactate formation is hypothesized to increase (see above), we found that plasma levels of glycerol remained unchanged during hibernation (Table 1 and Fig. 4) and, as previously reported [53], circulating lactate levels were lower in hibernating brown bears (Table 1). These results, together with the upregulation of liver gluconeogenesis-related genes seen previously in hibernating bears [54], strongly suggest that the uptake of glycerol and lactate by liver cells for fuelling the gluconeogenic pathway is sufficient to not only mask any increased release by peripheral tissues, but also to even be responsible for decreased circulating lactate levels. Alternatively, it could be that lactate levels were higher in summer-active bears, with the possible superimposed effect of the short run when the helicopter approached, as suggested in a previous study [53]. Concerning alanine, its decreased plasma levels in hibernating bears (Fig. 2) could be a combination of its uptake by the liver and a lower formation rate, due to decreased protein turnover [14]. In this regard, we also observed that alanine aminotransferase 1 (ALAT1) levels were reduced in bear muscles during winter, suggesting that alanine formation is affected during hibernation. Finally, liver gluconeogenesis could also be fuelled by amino acids produced from a certain degree of protein degradation, although atrophy remains limited. Gluconeogenesis can also be achieved by the kidneys, especially from lactate and glutamine as the main substrates [55]. Increased glutamine plasma levels in hibernating bears could, at least in part, be due to its lesser utilization by the kidney. This would agree with a reduction of kidney activity during hibernation [56]. This might also be related to bear urea nitrogen recycling during hibernation [57] since glutamine is the major amino acid in the blood that provides inter-organ transport of nitrogen (and ammonia).

The regulation of muscle enzymes of glycogen metabolism that we observed here suggests that muscle glycogen stores are dynamically regulated in hibernating animals. We actually found higher glycogen levels in the muscles of brown bears during hibernation than in summer (Fig. 5), similar to a recent report on American black bears [58], whereas only a non-significant trend toward such a seasonal change was observed in hibernating ground squirrels [59]. Although we cannot determine exactly when glycogen deposits are made into muscle, this could provide an easy source of glucose for muscle glycolysis during hibernation.

Beneficial effects on health are generally triggered by n-3 polyunsaturated fatty acids (n-3 PUFAs) and we suggest they could be extended to the hibernation state. Indeed, docosahexaenoic acid (DHA) has been reported to elevate glycogen stores [60] and enhance the expression of glucose uptake-related genes [61] in laboratory mouse muscles. DHA also appears to prevent fasting-induced muscle atrophy [60] and fight against sarcopenia [62], induce muscle protein synthesis [63] and prevent palmitate-induced proteolysis [64]. In an earlier study, we found no difference in levels of eicosapentaenoic acid (EPA) in skeletal muscle tissue of hibernating bears, but its plasma levels were decreased [65]. We extend these results here by showing the rise in circulating concentrations of docosapentaenoic acid (DPA) and DHA in hibernating compared to active brown bears (Fig. 5). Hence, the rise in DHA levels could mediate, at least in part, several of the bear muscle responses to hibernation that we have reported and discussed in this study.

## Conclusions

The objective of this study was to examine how energy and fuel metabolism are regulated in the skeletal muscle of hibernating brown bears. Through quantification of proteins involved in energy and fuel metabolism and using assays of enzyme activities, we found that bear muscle metabolism is dominated by reduced ATP turnover, with lipids being the preferred fuels, but their rate of oxidation being reduced during hibernation due to and/or contributing to metabolic rate depression. Concomitantly, glycolysis appears to be maintained and it could be fuelled from liver gluconeogenesis and the mobilization of large muscle glycogen stores, although we cannot conclude definitely on that point. Such metabolic regulations no doubt favour energy savings while maintaining hibernating bear muscle proteins, which may enable bears to react quickly in the case of unexpected disruption or early arousal from hibernation. A potential role of n-3 PUFAs could be hypothesized. To go further in understanding muscle metabolism during bear hibernation, mitochondria are the next target to explore using, e.g., high-resolution respirometry and evaluation of the response to various substrates.

## Methods

### Bear sample collection

Samples were collected from 37 free-ranging brown bears (*Ursus arctos*; 26 females and 11 males) from Dalarna and Gävleborg counties, Sweden, from 2011 to 2018. The animals were two (23 bears) or three (14 bears) years old. In a given year, the bears were captured during hibernation (February) and recaptured during their active period (June). The mean body mass of hibernating (45.9 ± 2.7 kg) versus summer active (42.9 ± 2.2 kg) bears was not significantly different (paired t-test, *p* = 0.06). Bear immobilization was performed as previously described [53, 66]. Blood samples were collected from the jugular vein within 20 min after darting, either in tubes containing a clotting activator (Vacuette® Z serum Sep Clot Activator, Greiner Bio-One GmbH, Kremsmünster, Austria) or in tubes with spray-coated K_2_EDTA (BD Vacutainer®, FisherScientific, Illkirch, France). Tubes were centrifuged (3000 g, 20 min) within 1 h after sampling and the serum or plasma was immediately frozen on dry ice until storage at − 80 °C. Biopsies of the vastus lateralis muscle were collected at the same time and immediately frozen on dry ice until storage at − 80 °C. The study was approved by the Swedish Ethical Committee on Animal Experiment (applications #C212/9, #C47/9, #C7/12, #C268/12, and #C18/15), the Swedish Environmental Protection Agency (NV-0758-14), and the Swedish Board of Agriculture (31-11,102/12). All procedures complied with Swedish laws and regulations.

Additionally, we obtained samples of skeletal muscle tissue (vastus lateralis) from two euthanized captive brown bears stored and provided by the Norwegian Veterinary Institute, and from 5 brown bears euthanized at Orsa Predator Park, Sweden (permit N° Dnr5.8.18-06068/2017). Upon euthanization, these seven bears were active, i.e. not hibernating, and are referred to as ‘summer captive’ animals in this study.

### Sample pre-processing for proteomics-based analyses

Frozen muscle bear biopsies (*N* = 7 per season) were pulverized using a laboratory ball mill (Mikrodismembrator, Sartorius). The resulting powders were used for running complementary proteomics approaches.

### Mass spectrometry-based quantitative analysis of bear muscle proteome

Ground muscle powders were suspended in a buffer composed of 8 M urea, 2 M thiourea, 4% Chaps, 1% dithiothreitol, 0.05% TLCK and 0.02 to 2 mM protease inhibitors. These were then sonicated (10 s, 135 watts) on ice, followed by addition of nine volumes of cold acetone; samples were then kept at − 20 °C overnight. Precipitated proteins were pelleted by centrifugation (10 min, 4 °C, 14000 g), and after discarding supernatants, the pellets were vacuum-dried (Speedvac, ThermoFisher Scientific; Rockford, IL, USA) and then dissolved in a buffer composed of 8 M urea, 2 M thiourea, 4% Chaps, 0.05% TLCK and 0.02-2 mM protease inhibitors in 30 mM Tris (pH 8.8). Protein concentration in protein extracts was measured using the Bradford protocol (Bio-Rad, Hercules, CA, USA). At this stage, one sample pool, comprising equal amounts of all muscle protein extracts, was generated for quality assessment of LC-MS/MS.

Aliquots of 10 μg of bear proteins (individual samples and sample pool) were precipitated with acetone (9 volumes) during 2 h at − 20 °C. After centrifugation (10 min, 15,000 g), protein pellets were solubilized in sample buffer (50 mM Tris pH 6.8, 1 mM EDTA, 5% β-mercaptoethanol, 2.5% SDS, 10% glycerol and 0.1% Bromophenol blue), and incubated at 95 °C for 5 min. Electrophoresis was performed using polyacrylamide gels. Sample preparation was performed in duplicate for each bear muscle extract. After colloidal Coomassie blue staining (G250, Fluka, Buchs, Switzerland), five protein bands (2 mm each) were excised from the gels. Proteins were in-gel reduced and alkylated using an automatic pipetting device (MassPrep, Waters) and digested overnight with trypsin (Promega, Madison, WI, USA) at an enzyme-to-protein-ratio of 1:100. Peptides were extracted in 60% acetonitrile/0.1% formic acid in water for 1 h, at 450 rpm on an orbital shaker. Organic solvent was finally removed in a Speedvac and samples were adjusted to 30 μl with 1% acetonitrile containing 0.1% formic acid.

Tryptic peptides (3 μL aliquots) were analysed on a UPLC-system (nanoAcquity, Waters, Milford, MA, USA) coupled to a quadrupole-TOF hybrid mass spectrometer (maXis 4G; Bruker Daltonik GmbH, Bremen, Germany). Injection of individual samples was performed in duplicate. The instrument was controlled by Bruker compass Hystar (v3.2) and OtofControl (Rev3.2). The solvent system consisted of 0.1% formic acid in water (solvent A) and 0.1% formic acid in acetonitrile (solvent B). Each sample was first concentrated/desalted on a trap column (Symmetry C18, 180 μm × 20 mm, 5 μm; Waters) at 1% B at a flow rate of 5 μl/min for 3 min. Afterwards, peptides were eluted from the separation column (BEH130 C18, 75 μm × 250 mm, 1.7 μm; Waters) maintained at 60 °C using a 60 min gradient from 8 to 35% B at a flow rate of 450 nl/min. The mass spectrometer was operated in positive mode, with automatic switching between MS and MS/MS scans. The source temperature was set to 160 °C with a spray voltage of − 4.5 kV and dry gas flow rate of 5 l/min. External mass calibration of the Tof (MaXis 4G) was achieved before each set of analyses using Tuning Mix (Agilent Technologies, Paolo Alto, USA) in the mass range of 322-2722 m/z, and mass correction was achieved by recalibration of acquired spectra to the applied lock mass using hexakis (2,2,3,3,-tetrafluoropropoxy)phosphazine ([M + H]^+^ 922.0098 m/z). The MS acquisition time was set to 0.4 s, and MS/MS acquisition time to a range from 0.05 s (intensity > 250,000) to 1.25 s (intensity < 5000), and ions were excluded after acquisition of one MS/MS spectrum with release of exclusion after 0.2 min. Up to 10 most intense multiply charged precursors per MS scan were isolated, using an isolation window adapted to the isolated m/z (2-5 m/z), then fragmented using energy collisional dissociation. The sample pool (quality control) was repeatedly analyzed throughout the whole duration of these experiments to enable verification of the stability of the nanoLC-MS/MS system and reproducibility of the quantitative measurements.

MS raw data were processed using MaxQuant (v1.5.3.30) [67]. Peak lists were searched using the decoy mode (revert) of the Andromeda search engine implemented in MaxQuant against a protein database created using the MSDA software suite [68]. The database contained bear protein sequences (UniprotKb; Taxonomy ID: 9632; downloaded in September 2017) to which sequences of common contaminants (e.g. trypsin and keratins) were automatically added via MSDA and MaxQuant. Regarding search parameters, MS tolerance was set to 0.07 Da for the first search and 0.006 Da for the main search. MS/MS tolerance was set to 40 ppm. A maximum number of two missed cleavages was accepted, and carbamidomethylation of cysteine residues was set as fixed modification, while acetylation of protein N-termini and oxidation of methionine residues were set as variable modifications. False discovery rates (FDR) were set to 1% for both peptide spectrum matches (minimum length of 7 amino acids) and proteins. The final list of identified proteins did not consider common contaminants, which were removed. Regarding quantification, data normalization and estimation of protein abundance was performed using the MaxLFQ (label free quantification) option implemented in MaxQuant [67]. MaxLFQ quantification was applied using a minimal ratio count of two. Both unmodified and modified (acetylation of protein N-termini and oxidation of methionine residues) peptides were considered for quantification. All other MaxQuant parameters were set as default. After having checked the reproducibility of technical replicates, their median value was calculated for each individual sample. Then, only proteins with at least five of seven values per season (i.e. accepting a maximum of 2 missing values) were retained for further analysis, as well as “present/absent” cases (i.e. 0 values in the samples from one of the two seasons). A total of 725 bear muscle proteins were identified robustly, of which 538 fulfilled the above-mentioned criteria (Additional file 1: Table S1). To retain only the proteins with a highly reproducible quantification, we compared replicate injections and then replicate preparations of individual samples and 383 proteins were found to comply with such stringency.

Regarding quality controls, we considered only peptides detected in at least eight of the ten repeated injections of the sample mixes. The mean coefficient of variation (CV) of retention times of the peptides of glyceraldehyde-3-phosphate dehydrogenase, chosen as a common housekeeping protein, was 3.3%. In addition, the median coefficient of variation for LFQ values from all proteins across repeated injections of sample mixes was 25%. These different values indicate good stability of the nanoLC-MS/MS system over the whole duration of the analyses, and also good reproducibility of protein abundance determination.

### Gel-based quantitative analysis of bear muscle proteome

Ground muscle powders were dissolved in a denaturing solution (urea 7 M, thiourea 2 M, Chaps 4%, DTT 1%, protease inhibitors 0.02 to 2 mM, TLCK 0.0005%) and protein extraction was performed by incubation one hour at room temperature followed by sonication on ice (10 s., 135 W). Proteins were then acetone-precipitated overnight at − 20 °C using nine volumes of cold acetone and pelleted by centrifugation (15 min, 14,000 g, 4 °C). After vacuum-drying using a Speedvac ThermoFisher Scientific; Rockford, IL, USA), proteins were dissolved in a solution composed of 7 M urea, 2 M thiourea, 30 mM Tris (pH 8.5) and 4% Chaps. The pH was then adjusted to 8.5, and homogenization was completed by sonication on ice (10 s, 135 watts). After removing cell debris by centrifugation and collection of the supernatant, soluble protein concentrations were determined using the Bradford protocol (Bio-Rad). For each sample, protein profiles were checked on 12% SDS-PAGE acrylamide gels (10 μg loaded; 50 V for 60 min and then 100 V to complete migration) using Coomassie brilliant blue (Sigma Aldrich; St. Louis, MO, USA) staining. The similarity of protein profiles between all samples was then verified prior to quantitative DIGE analysis.

Protein samples were labelled using a CyDye DIGE Fluor Minimal Dye Labeling Kit (GE HealthCare, Uppsala, Sweden). CyDyes were first reconstituted in anhydrous N,N-dimethylformamide, then 400 pmol of Cy3 and Cy5 were used to randomly label 50 μg of protein samples from the different groups, and Cy2 (1.2 nmol/150 μg proteins) was used to label a mixture of all the samples (25 μg each) that was used as an internal standard. After incubation in the dark for 30 min on ice, protein labelling was quenched by addition of 10 mM of lysine stop solution and incubation in the dark for 10 min on ice.

Prior to 2D gel electrophoresis, the multiplexing of samples from hibernating and active bears was randomized to avoid any bias. Briefly, 50 μg of Cy2, Cy3 and Cy5-labelled protein samples were mixed and diluted with 7 M urea, 2 M thiourea, 2% Chaps, 2% DTT, 2% ampholytes (GE Healthcare), protease inhibitors and a trace of bromophenol blue. Proteins were then loaded onto 24 cm pH 3-10 non-linear immobilized pH gradient strips (IPG Ready strip, Biorad, Hercules, CA, USA), and left in the dark for passive rehydration over 1 h. Active rehydration was then performed in the dark over 15 h at 50 V using a Protean IEF cell (Biorad). Isoelectric focusing was performed using voltage gradient steps (from 0 to 200 V over 1 h, from 200 to 1000 V over 4 h, from 1000 to 5000 V over 16 h, then 5000 V for 7 h; total of 90000Vh). Focused proteins were then reduced by incubation (15 min) of IPG strips in a solution composed of 1% DTT, 6 M urea, 50 mM Tris pH 8.8, 30% glycerol and 2% SDS. Proteins were alkylated by incubation (15 min) in a solution composed of 2.5% iodoacetamide, 6 M urea, 50 mM Tris pH 8.8, 30% glycerol and 2% SDS. Electrophoresis was carried out using 12.5% polyacrylamide gels (2DGel DALT NF; Serva Electrophoresis GmbH, Heidelberg, Germany) and a HPE FlatTop Tower (Gel Company, CA, USA). Electrophoresis began with application of 7 mA per gel for 30 min followed by 13 mA per gel for 30 min, then 20 mA per gel for 10 min, 40 mA per gel for 4 h and, finally, 45 mA per gel for 40 min.

Following 2D gel electrophoresis, gels were washed with water and gel images were acquired using an Ettan DIGE Imager (GE HealthCare) at 100 μm resolution. Gel images were analysed using the Progenesis Samespots software (v4.5; Nonlinear Dynamics, Newcastle upon Tyne, UK). After automatic alignment, the quality of spot matching was checked and, when necessary, improved by application of minor manual corrections. After background subtraction and normalization of Cy3 and Cy5 spot volumes to that of corresponding Cy2 spots, the spots highlighted as differential by statistical analysis were excised. Three to four matched spots were put together, and proteins were in-gel reduced and alkylated using a Massprep Station (Waters, MicroMass, Manchester, UK) as previously reported [69]. For in-gel digestion of proteins, 25 μL of a 12.5 ng/L trypsin (Promega) solution in 25 mM NH_4_HCO_3_ were added to gel pieces before incubation for 12 h at 37 °C. The resulting peptides were extracted using 30 μL of a 60% acetonitrile (Carlo Erba, Val de Reuil, France) solution containing formic acid (0.1%). Acetonitrile was removed by vacuum drying using a Speedvac.

Tryptic peptides were analyzed on a UPLC-system (nanoAcquity, Waters) coupled to a quadrupole-TOF hybrid mass spectrometer (maXis 4G; Bruker Daltonik GmbH, Bremen, Germany), exactly as reported above for label-free (XIC) MS-based analyses, except from the elution gradient (t = 0 min 1% B, t = 9 min 35% B, t = 10 min, 90% B) and the fact that isolation and fragmentation was performed here for the 6 most intense multiply charged ions, with exclusion being set to 1 min.

MS/MS data were analysed using the Mascot™ search engine (v2.5.1, Matrix Science, London, UK) installed on a local server. Spectra were searched against a target-decoy version of the same bear protein database that was used for label-free (XIC) MS1-based analyses (see above), to which sequences of common contaminants (e.g. trypsin and keratins) were automatically added using the MSDA software suite [68]. Mascot search parameters included a mass tolerance of 10 ppm in MS and 0.05 Da for MS/MS modes, a maximum of one trypsin missed cleavage allowed, carbamidomethylation of cysteine residues set as fixed modification, and oxidation of methionine residues and acetylation of protein N-termini set as variable modifications. Stringent filtering criteria were applied using Proline software (v2.5.1; http://proline.profiproteomics.fr/) to obtain high-confidence identifications (FDR < 1% at both protein set and PSM level; and a minimal PSM score of 25). Single-peptide-based protein identifications, as well as the identification of common contaminants such as keratin and trypsin, were not considered.

Among the different proteins that were identified in a given spot, only the major (more abundant) ones were considered to be responsible for variations of spot intensities. The determination of major proteins was performed following a “peptide counting” strategy: the higher the number of peptides assigned to a given protein, the more abundant this protein is. We took into account the fact that tryptic sites are followed or not by a proline residue, the possible missed cleavages and the adequate size of peptides for detection by mass spectrometry (i.e., based on our data, peptides of 7 to 32 amino acids. The theoretical number of detectable tryptic peptides was similar for major and minor proteins in a given gel spot, as their ratio was of 1.0 ± 0.1. However, major proteins were identified with an experimental number of peptides 4.1 ± 1.6 times higher than minor ones.

### Quantitative RT-PCR analyses in bear muscle

Total RNA was isolated from bear muscle (*N* = 8 per season) using TRIzol reagent (Invitrogen, Courtaboeuf, France) according to manufacturer’s instructions. First-strand cDNAs were synthesized from 1 μg of total RNA using PrimeScript RT kit (Ozyme, Saint Quentin en Yveline, France) with a mixture of random hexamers and oligo(dT) primers and treated with 60 units of RNaseH (Ozyme). Real-time PCR assays were performed with Rotor-Gene 6000 (Qiagen, Courtaboeuf, France). Different primers were used for fatty acid translocase (CD36; forward: 5′-CAGACAGTTTTGGATCTTTG-3′; reverse: 5′-CTGGGTTACATTTTCCTTGG-3′), aquaporin 7 (AQP-7; forward: 5′-AGATGGTGCGAGAGTTCCTG-3′; reverse: 5′-TGACTCCGAAGCCAAAACCC-3′), monocarboxylate transporter 1 (MCT1 or SLC16A1; forward: 5′-GTTGGTGGCTGCTTGTCAGG-3′; reverse: 5′-TTCAAGTTGAAGGCAAGCCC-3′) and monocarboxylate transporter 4 (MCT4 or SLC16A3; forward: 5′-AGCTCATGCGTGAGTTTGGG-3′; reverse: 5′-CCAAAGCGGTTCACGCACAC-3′). The results were normalized to mRNA levels of TATA box binding protein (TBP; forward: 5′- AGACCATTGCACTTCGTGCC-3′; reverse: 5′-CCTGTGCACACCATTTTCCC-3′) used as a reference gene in each sample.

### Western-blot analyses in bear muscle

Proteins were extracted from bear muscle (*N* = 12 per season) using ice-cold lysis buffer (Tris-HCl 20 mM, NaCl 138 mM, KCl 2.7 mM, MgCl_2_ 1 mM, glycerol 5%, NP 40 1%, EDTA 5 mM, Na_3_VO_4_ 1 mM, NaF 20 mM and DTT 1 mM) supplemented with protease inhibitor cocktail (Sigma Aldrich, St Louis, MO, USA). Homogenization was performed using a Precellys homogenizer (Bertin Instruments, Montigny-le-Bretonneux, France) with three shaking cycles of 30 s at 6800 rpm followed by 5 min at 4 °C. After centrifugation (15 min, 12,000 g), protein concentration in the supernatant was determined by Bradford quantification.

Western blotting was performed as previously described [70]. After loading 20 μg of total protein into each well of SDS-PAGE gels, electrophoresis and electroblotting to PVDF membrane were carried out. Membranes were blocked with 4% BSA (Bovine Serum Albumin, Euromedex, Souffelweyersheim, France) and then probed with primary antibodies purchased from Abcam (rabbit anti-CD36: #ab133625; mouse cocktail anti-OXPHOS [oxidative phosphorylation]: #ab110413; both used at 1/1000), and Santa Cruz Biotechnology (rabbit anti-HADH: #sc292196; goat anti-citrate synthase: #sc242444; both used at 1/1000). Corresponding secondary HRP antibodies from BioRad (goat anti-rabbit IgG (H + L)-HRP conjugate: #1721019; goat anti-mouse IgG (H + L)-HRP conjugate: #1721011; rabbit anti-goat IgG (H + L)-HRP conjugate: #1721034; all used at 1/10000) were used for chemiluminescence visualization (Chemidoc; Bio-Rad, Hercules, CA, USA).

### Glycogen content and enzyme activity measurements in bear muscle

Glycogen content, pyruvate kinase (PK), and lactacte dehydrogenase (LDH) activities were quantified in bear muscle (*N* = 7 per season) using commercial kits (#MAK016, #MAK072 and #MAK066, respectively) from Sigma Aldrich. Assays were performed according to manufacturer’s instructions, using 20, 3 and 0.3 μg of total protein, respectively.

Citrate synthase (CS) and cytochrome c oxidase (COX) activities were measured as described previously [71], using 10 μg of total protein. β-Hydroxyacyl-CoA dehydrogenase (HAD) activity was measured in the same homogenates using 10 μg of protein. The reaction was performed in 1 ml of reaction buffer containing HEPES 20 mM, EGTA 1 mM, KCN 1 mM, and NADH 0.15 mM, pH = 7.4 at 25 °C. Assays were started by the addition of 0.1 mM acetoacetyl-CoA and the oxidation of NADH was measured at 340 nm.

### Metabolite assays and amino acid measurements in bear plasma

Levels of metabolites and amino acids were assessed in bear plasma (*N* = 6-8 per season, except from glycerol assays performed in *N* = 25 per season). Plasma free fatty acids and lactate were determined enzymatically using kits from Randox Laboratories Ltd. (Crumlin, UK), and glycerol, glucose, and triacylglycerol levels were assessed using kits from Cayman Chemical (Ann Arbor, MI, USA), Sigma Diagnostics (St. Louis, MO, USA) and Biolabo SAS (Maizy, France), respectively.

After deproteinization of 250 μL of plasma with sulfosalicylic acid (Sigma Diagnostics), circulating amino acid concentrations were determined by ion-exchange chromatography followed by a postcolumn derivatization with ninhydrin (Hitachi L8900, ScienceTec, Villebon-sur-Yvette, France).

### NMR-based analysis of bear plasma metabolome

For ^1^H NMR profiling, 100 μL of each plasma sample (N = 7 per season) was first evaporated using a vacuum concentrator (SpeedVac, ThermoFisher Scientific) and further dissolved in 100 μL of D_2_O (99.96% minimum; Eurisotop) and 100 μL of 300 mM phosphate buffer (KH_2_PO_4_ and K_2_HPO_4_; pH 7.06; Fluka). The final analytical sample (200 μL) contained 10% phosphate buffer (30 mM) and TSP–d4 (3-(trimethylsilyl) propionic-2,2,3,3- tetra-d4 acid sodium salt 0.5 mM, 98% deuterated; Sigma-Aldrich) in D_2_O was filled in Shigemi 5 mm symmetrical NMR microtube assembly (Sigma-Aldrich).

For 2D NMR identification, all the previous samples were pooled, then evaporated and further dissolved in 600 μL D_2_O before filling a 5 mm NMR tube (Norell, Eurisotop). D_2_O was used for shimming and locking, whereas TSP-d_4_ constituted a reference for chemical shifts (0 ppm) for ^1^H NMR.

One-dimensional ^1^H NMR experiments (i.e. profiles) were conducted on a Bruker Avance 500 MHz equipped with a 5-mm inverse-triple tuned (TXI) ^1^H/1^3^C/^15^N with z-gradient coil probe (Bruker Biospin Wissenbourg, France). For each profile, two standard monodimensional sequences were acquired. The nuclear overhauser effect spectroscopy sequence (noesygppr1d with water presaturation and gradients) and the Carr-Purecell-Meiboom-Gill sequence (cpmgpr1d with water presaturation) were used with low power irradiation of the water resonance during the recycle delay of 4 s and the mixing time of 10 ms. For the cpmgpr1d sequence the echo time was fixed to 70 ms. For all the spectra, 512 scans were collected with a 90° impulsion time of 9.75 μs, an acquisition time of 3.3 s, a spectral window of 10,000 Hz and 64 K data points zero-filled to 128 K before Fourier transformation with 0.3 Hz line broadening. Spectra were treated with Topspin version 3.1. All NMR spectra were recorded at 300 K. For the identification of metabolites in the pooled sample, we used the NMR spectrometer at the TGIR NMR Facility (TGIR-RMN-THC Fr3050 CNRS- Gif/Yvette, France), a Bruker Avance III 950 MHz equipped with a 5 mm TCI (^1^H/1^3^C/^15^N/^2^H) cryoprobe with z-gradient coil probe (Bruker Biospin). For 1D ^1^H-Spectra, a standard one dimensional spectroscopy sequence (noesygppr1d) was used with low power irradiation of the water resonance during the recycle delay of 4 s and a mixing time of 10 ms. For each spectrum, eight scans were collected with a 90° impulsion time of 7.7 μs, an acquisition time of 3.3 s, a spectral window of 10,000 Hz and 64 K data points zero-filled to 128 K before Fourier transformation with 0,3 Hz line broadening. All 2D homonuclear (^1^H-^1^H COSY, ^1^H-^1^H TOCSY, ^1^H-^1^H JRES) and heteronuclear (^1^H-^13^C HSQC and HMBC) experiments were performed with quadrature phase detection in dimensions, using state-TPPI or QF detection mode in the indirect one. For each 512 increments in the indirect dimension, 2 K data points were collected and 32 or 64 transients were accumulated in the direct dimension. A ^13^C decoupling (GARP) was performed during acquisition time for heteronuclear experiments. A π/2 shifted square sine-bell function was applied in the both dimensions before Fourier transformation. Spectra were treated with Topspin version 3.5pl5. All NMR spectra were recorded at 300 K.

We divided 1D ^1^H-NMR spectra into 10,000 regions (buckets) of 0.001 ppm wide. After reduction over the chemical range of − 0.5 to 10 ppm and considering an exclusion area around the residual water signal (4.6 to 5.1 ppm), bucketing was performed using the AMIX software (Bruker GmbH). The signal intensity in each bucket was integrated using the sum intensities mode and spectra were scaled to total intensity. From Noesy and CPMG spectra, the buckets with a VIP score above 1, as revealed by PLS discriminant analysis (PLD-DA), were analyzed deeply (1d and 2d NMR) in order to identify the related metabolites. Identification of the metabolites (VIP buckets) based on the 1D and 2D NMR spectra was done using metabolite databases HMDB [72], BMRB [73] and relevant publications [74–77].

### Imaging of bear muscle fibers using electron microscopy

Freshly collected muscle biopsies were fixed immediately with 2.5% glutaraldehyde (Electron Microscopy Sciences, Hatfield, PA, USA), then washed using PBS and post-fixed in 1% osmium tetroxide (OsO4; Sigma Aldrich). After dehydration in ethanol, followed by incubation in propylene oxide (VWR, Radnor, PA, USA), samples were embedded in Epon (Electron Microscopy Sciences) and kept at 60 °C overnight for polymerization. Ultrathin sections were cut with a Reichert Ultracut S ultramicrotome, stained with uranyl acetate and lead citrate (Merck, Darmstadt, Germany), and examined with a transmission electron microscope (JEOL 1011). Digital images were obtained using GATAN CCD Orius 1000 camera with Digital Micrograph software.

### Lipidomics analysis of bear serum

We prepared eight different mixes from individual bear sera collected during four successive years (2012-2015), i.e. one per season per year. Serum mixes consisted of equivalent volumes of 2-7 individual samples collected in a given season and year. Total lipids from bear serum mixes (*N* = 4 per season) were extracted and analyzed, as previously described [78]. Briefly, a double extraction was carried out using ethanol/chloroform (1:2, *v*/v) after internal standard addition. The organic phases were dried under nitrogen, and samples were treated with toluene-methanol (1:1, v/v) and boron trifluoride in methanol (14%). Lipid transmethylation was then carried out at 100 °C for 90 min in screw-capped tubes. After the addition of 1.5 mL of K_2_CO_3_ in 10% water, fatty acid methyl esters were extracted using 2 mL of isooctane and analyzed by gas chromatography (HP6890 instrument equipped with a fused silica capillary BPX70 SGE column; 60 × 0.25 mm). The vector gas was hydrogen. Temperatures of the Ross injector and flame ionization detector were set to 230 °C and 250 °C, respectively.

### Statistical analysis

Multiple bears were analyzed as biological replicates. Statistical analysis was performed using the R software environment v3.0.2 [79]. Shapiro-Wilk, Bartlett, Welch two sample and paired student t-tests, one-way ANOVA and Tukey tests used the FactoMineR package. Type III ANOVA used several R packages, including lmerTest, lme4, lsmeans, and multcomp. It should be noted that adjustment of *p*-values for multiple testing was done using either the Benjamin-Hochberg (t-tests) or the Bonferroni (ANOVA and Tukey tests) method.

For label-free (XIC) MS1-based analyses, the reproducibility of MaxLFQ values among technical replicates was first checked using one-way ANOVA (*p* > 0.05). For both proteomics approaches, we used the Shapiro-Wilk test (p-value > 0.01) to check the normal distribution of MaxLFQ values (XIC) and of normalized spot volumes (2D-DIGE). Homoscedasticity was then tested using the Bartlett test (*p*-value > 0.01). Changes in the abundance of proteins between hibernating and summer-active bears were assessed using Welch two sample t-tests for both proteomics approaches or paired student t-tests only for the 2D-DIGE approach, significance being set to *p*-values < 0.05. Paired student t-tests were not applicable to XIC-derived data because of missing values.

For quantitative RT-PCR and western-blot analyses, as well as metabolites measured using kits, and for amino acid and fatty acid levels, we compared winter and summer seasons using paired student t-tests, with significance being set to *p*-values < 0.05.

For glycogen levels, we used one-way ANOVA followed by post-hoc Tukey tests to compare values in free-living and captive bears. Significance was set to p-values < 0.05 for both ANOVA and Tukey tests.

For enzyme activity measurements, a comparison of seasonal effects at different temperatures used a general linear-mixed effects model (Type III ANOVA with *p*-values for F-tests based on Satterthwaite method and post-hoc Tukey tests for multiple pairwise comparisons); significance was set to *p*-values < 0.05.

Statistical analysis of NMR-derived data was performed using SIMCA 13.0 software. Data matrices were filtrated using the Wilcoxon signed-rank test. A total of 96 buckets from NOESY spectra and 103 from CPMG spectra remained for further multivariate analyses. PCA did not reveal any outliers, and PLS-DA (according to the seasons; Pareto scaling) showed one principal component (Q^2^cum = 0.797) for NOESY data and one (Q^2^cum = 0.766) for CPMG data. Validations were made through “900 random permutations” and “observed vs. predicted”. PLS-DA showed VIP scores (i.e. importance into the model) for all buckets. On NOESY data, 11 buckets had a VIP score above 1.5 and 23 buckets above 1. On CPMG data, 11 buckets had a VIP score above 1.5 and 25 buckets above 1. Those buckets were analyzed deeply (1d and 2d NMR) in order to identify the related metabolites. Comparison of the relative abundance of identified metabolites in winter versus summer was performed using paired student t-tests, considering significance with *p*-values < 0.05.

### Bioinformatics analysis of proteomics data

Hierarchical clustering of proteomics data was performed using Cluster v3.0 software [80]. Parameters were set as follows: median centering and normalization of genes for adjusting data and centroid linkage clustering for both genes and arrays. Dendrograms were generated and viewed using the Treeview v1.6.6 program [81].

From the list of identified bear proteins, and in order to benefit from the more complete annotation of the human proteome, Swissprot identifiers of human protein homologues were retrieved using BLAST searches (FASTA program v36; downloaded from http://fasta.bioch.virginia.edu/fasta_www2/fasta_down.shtml). Only the best hits were retained, with a minimal sequence similarity of 78% for differential proteins, and their adequacy was checked manually for all proteins. Enrichment and functional annotation analysis of proteomics data was performed using the desktop version of DAVID (Ease v2.1) and an updated version of Gene Ontology (GO) databases (October 2017). Enriched GO terms were filtered by only considering those with an Ease score lower than 0.1, a Benjamini p-value lower than 0.01, and a fold enrichment higher than 2. Enriched GO terms were grouped together into broad functional categories, which were then considered as enriched broad functions.

## Additional files

Additional file 1: **Table S1.** List of vastus lateralis proteins analysed in hibernating (winter) and active (summer) brown bears (*Ursus arctos*) using a MS-based strategy. (XLSX 4373 kb)

Additional file 2: **Table S2.** List of vastus lateralis proteins identified in 2D gel spots, and exhibiting a differential abundance between hibernating (winter) and active (summer) brown bears (*Ursus arctos*). (XLSX 53 kb)

Additional file 3: **Figure S1.** Representative 2D gel image of muscle tissue proteins in bears. (PDF 183 kb)

Additional file 4: **Figure S2.** Representative blots of muscle tissue proteins in brown bears. (PDF 384 kb)

## Abbreviations

2D-DIGE: Two-dimensional differential in-gel electrophoresis
BLAST: Basic local alignment search tool
DHA: Docosahexaenoic acid
DPA: Docosapentaenoic acid
DTT: Dithiothreitol
EPA: Eicosapentaenoic acid
GO: Gene ontology
IPG: Immobilized pH gradient
MS: Mass spectrometry
MS/MS: Tandem mass spectrometry
MSDA: Mass spectrometry data analysis
NMR: Nuclear magnetic resonance
OXPHOS: Oxidative phosphorylation
PCR: Polymerase chain reaction
PUFA: Polyunsaturated fatty acids
RQ: Respiratory quotient
TOF: Time-of-flight
UPLC: Ultra performance liquid chromatography
XIC: Extracted ion chromatogram
