# Supplementary material for: Metabolic reprogramming involving glycolysis in the hibernating brown bear skeletal muscle
Source: Front Zool. 2019 May 6;16:12. doi: 10.1186/s12983-019-0312-2 (PMC6503430; doi:10.1186/s12983-019-0312-2)
Supplement: Supplementary file 3 — Figure S1. Representative 2D gel image of muscle tissue proteins in bears. (PDF 183 kb) [file 12983_2019_312_MOESM3_ESM.pdf]

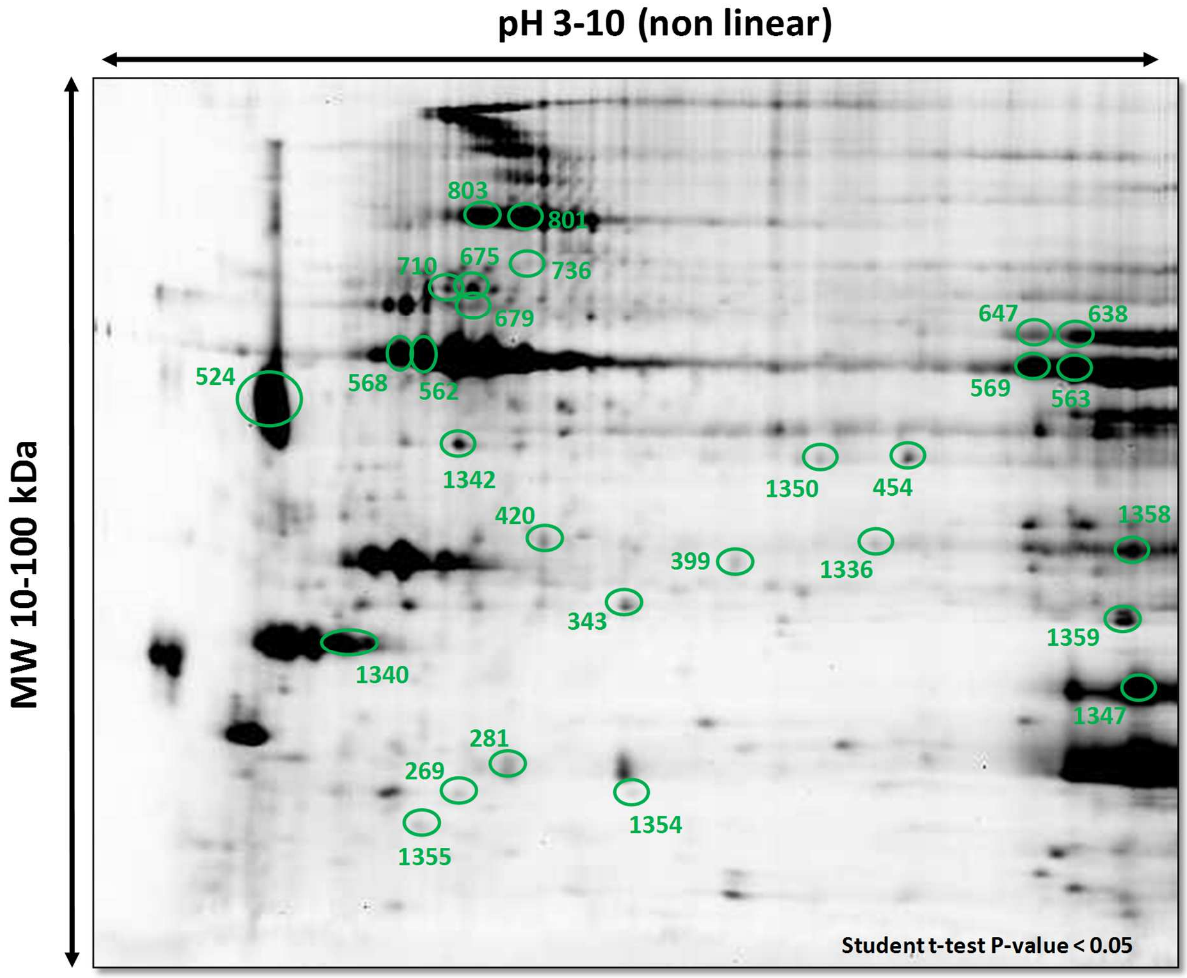

Figure S1. Representative 2D gel image of muscle tissue proteins in bears

Shown are the protein spots exhibiting significantly changed intensity between active (summer) and hibernating (winter) bears at Student t-test p-values < 0.05. Detailed image analysis information and mass spectrometry-based identification of the proteins contained in proteins spots can be found in Supplementary Table 2.
