# Supplementary material for: Metabolic reprogramming involving glycolysis in the hibernating brown bear skeletal muscle
Source: Front Zool. 2019 May 6;16:12. doi: 10.1186/s12983-019-0312-2 (PMC6503430; doi:10.1186/s12983-019-0312-2)
Supplement: Supplementary file 4 — Figure S2. Representative blots of muscle tissue proteins in brown bears. (PDF 384 kb) [file 12983_2019_312_MOESM4_ESM.pdf]

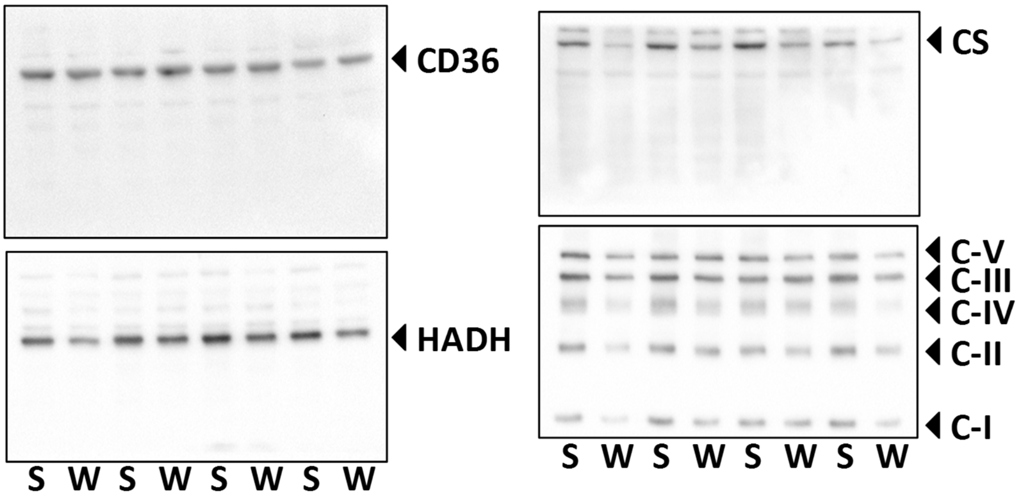

Figure S2. Representative blots of muscle tissue proteins in brown bears.
Shown are representative images that have been obtained through western-blot analysis of the levels of fatty acid translocase (CD36), mitochondrial hydroxyacyl-coenzyme A dehydrogenase (HADH), citrate synthase (CS) and subunits of the five OXPHOS complexes (C-I to C-V) in bear vastus lateralis muscles (n = 12/ season). Corresponding quantifications can be seen in Figure 3. S: Summer; W: Winter.
